# Antiobesogenic and Antiatherosclerotic Properties of *Caralluma fimbriata* Extract

**DOI:** 10.1155/2010/285301

**Published:** 2010-12-28

**Authors:** Soundararajan Kamalakkannan, Ramaswamy Rajendran, Ramasamy V. Venkatesh, Paul Clayton, Mohammad A. Akbarsha

**Affiliations:** ^1^Department of Animal Science, Bharathidasan University, Tiruchirappalli 620 024, India; ^2^Research and Development, GreenChem, Bangalore 562 107, India; ^3^Quality Control, Gencor Pacific, Hong Kong; ^4^Oxford Brookes University, Oxford OX3 0BP, UK; ^5^Mahatma Gandhi-Doerenkamp Center for Alternatives, Bharathidasan University, Tiruchirappalli-620 024, India

## Abstract

There is evidence that the principles present in the widely consumed Indian food plant *C. fimbriata* extract (CFE) suppress appetite, and provide antiobesogenic and metabolic benefits. The Diet-Induced Obesity (DIO) rat model was used to investigate CFE's anorexigenic effects. Rats were randomly divided into three groups: (i) untreated control (C), (ii) control for cafeteria diet (CA), and (iii) cafeteria diet fed + CFE treated. Rats in the test group received cafeteria diet and CFE from day one onwards. CFE was administered by gavage at three doses (25, 50, 100 mg/Kg BW per day) for 90 days. The antiobesogenic effects of CFE were evaluated by monitoring changes in feed intake, body weight, serum lipid and hormonal (leptin) profiles, fat pads, and liver weight. Antiatherosclerotic effects were measured by histology. CFE induced significant and dose-dependent inhibition of food intake, with dose-related prevention of gains in body weight, liver weight, and fat pad mass. Alterations in serum lipid profiles associated with weight gain were similarly inhibited, as were the typical increases in serum leptin levels. These data substantiate CFE's reported anorexigenic effects. CFE treatment also conferred protection against atherogenesis. We conclude that CFE possesses antiobesogenic and antiatherosclerotic properties.

## 1. Introduction

Obesity is increasingly prevalent. Cheap, calorie-dense foods and relatively inactive lifestyles create long-term imbalances between energy uptake and expenditure, leading to adipocyte hypertrophy and subsequently hyperplasia. Excess adipose tissue functions as an endocrine organ, producing bioactive molecules such as Il-6, TNF-alpha, and PAF-1 which are implicated in many disease states including diabetes and atherosclerosis. It also produces the peptide hormone leptin, which transmits a satiety signal to the hypothalamus and acts as a negative feedback loop of the lipostat [1]. Leptin effects on food intake and neuroendocrine functions involve intermediate hypothalamic neuropeptides such as proopiomelanocortin (POMC) and neuropeptide-Y (NPY) in the hypothalamus which regulate appetite, energy expenditure, and sympathetic nervous system outflow [1–3]. In obesity, the commonly found high leptin levels are not associated with appetite suppression, giving rise to the concept of central leptin resistance or insensitivity [4, 5]. This resistance may be caused by mutations affecting leptin transport, receptor affinity, or signal transduction. The majority of cases, however, probably reflect physiological de-sensitisation caused by excessive adipose tissue and leptin levels [6]. Restoring leptin sensitivity and hypothalamic appetite control presents an exciting new target in obesity management. 

Pharmaceutical approaches are still in their early stages. The pregnane glycosides in Hoodia [7] exert appetite-suppressant effects via enhanced hypothalamic signaling [8], but this plant risks extinction. We studied *Caralluma fimbriata*, a traditional Indian “famine food” with no history of adverse effects, which also contains pregnane glycosides [9]. This study evaluated the extract of *C. fimbriata *(CFE, Slimaluma.) for appetite suppressing, antiobesogenic and antiatherogenic properties in the DIO rat model.

## 2. Materials and Methods

### 2.1. Animals and Housing

Male Wistar rats (200–220 g), bred from a stock obtained from the Central Animal Facility, Indian Institute of Science, Bangalore, India, were used in the study. Animals were housed individually in polypropylene cages for at least 1 week of acclimation before the experiment started. Animals were cared for in accordance with the principles of animal ethics guidelines, and the study was approved by the Institutional Animal Ethics Committee (IAEC). The rats were maintained under standard laboratory conditions at a room temperature at 23 ± 1°C with relative humidity: 50 ± 10% and 12 h light/dark cycle. All experiments were conducted under strictly controlled and pathogen-free conditions.

### 2.2. Procedures

The *C. fimbriata* extract (CFE) was prepared and gifted by GreenChem, Bangalore, India. To produce CFE, the aerial parts of the plant were extracted with alcohol to obtain a 25% solution of pregnane glycosides, which was lyophilized to powder.

### 2.3. Experimental Design

The animals were randomly divided into three groups (*N* = 6): (i) untreated control, (ii) control for cafeteria diet (CA), and (iii) cafeteria diet fed + CFE treated. Rats in the untreated control group were fed standard pellet chow ad libitum, while rats in the CA and CA + CFE treatment groups received both pellet chow and cafeteria diet. CFE was administered by gavage, at three different doses, 25, 50, 100 mg/kg/day, for 90 days.

### 2.4. Diet-Induced Obesity (DIO)

Obesity was induced by providing modified versions of the Harris (1993) high-fat cafeteria diet [10]. It consisted of 3 variants: (i) condensed milk + bread + peanuts + pellet chow (4 : 1 : 4 : 1), (ii) chocolate + biscuits + dried coconut + pellet chow (3 : 2 : 4 : 1), and (iii) cheese + boiled potatoes + beef tallow + pellet chow (4 : 2 : 4 : 1). The different variants were presented on alternate days throughout the treatment period.

### 2.5. Indirect Antiappetite Assay

Group (iii) animals received cafeteria diet and CFE from day one. Daily food consumption was determined by weighing feed clearance. The appetite suppressing activity of CFE was calculated by monitoring (a) food intake for 90 days, (b) animal's bodyweight at baseline, weekly and at term, and (c) the weights of fat pads (perirenal, mesenteric, and,epididymal) and liver at term.

### 2.6. Analysis of Serum Obesity Indicators

Serum obesity indicators including cholesterol (TC), triglycerides (TG), and high-density lipoprotein (HDL) were measured using a commercial kit (Span Diagnostic Ltd, India) in semiautomatic analyzer (Micro Lab-200). Low-density lipoprotein (LDL) and very low-density lipoprotein (VLDL) levels were calculated adopting the prescribed formula.

### 2.7. Analysis of Serum Leptin

At term, blood was collected by cardiac puncture under mild sodium pentothal anesthesia; the serum was separated and stored at −20°C until analysis. Leptin was determined using a commercial rat Enzyme-Linked Immunosorbent Assay (ELISA) kit (BioVendor, Czech Republic).

### 2.8. Antiatherosclerosis Study

Animals were initially perfused with saline. Sections of aorta from aortic arch to thoracic aorta were removed and fixed in 10% formalin neutrally buffered solution. 3 mm segments taken at 5 mm distance from the bifurcation of the left subclavian artery were embedded in paraffin wax. 5 *μ*m thick cross-sections were cut and stained with hematoxylin and eosin to determine intimal thickening.

### 2.9. Data Analysis

All the values were expressed as the mean ± SEM and analyzed by one-way analysis of variance (ANOVA) using the Brown-Forsythe statistic followed by Games-Howell post hoc comparisons tests in order to test differences between groups. These options were chosen as they do not require any assumptions of homogeneity of variance. The level of statistical significance was set at *P* < .05. The statistical analyses were run using SPSS for Windows (version 16, Chicago, IL).

## 3. Results

### 3.1. Feeding Behavior

**Figure 1:**
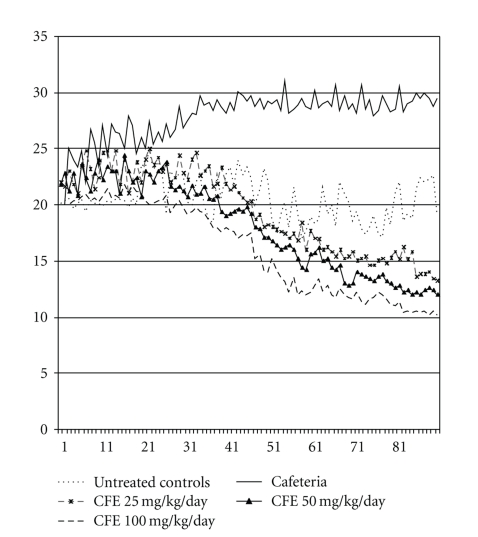
Feed intake in grams over 90 days.

Feeding behavior was monitored in the test animals for 90 days (Figure 1). As expected, food intake was significantly greater in CA-fed groups than in the group given pellet chow (*P* < .05). Concurrent administration of 25, 50, and 100 mg/kg/day of CFE with CA reduced food intake considerably compared to both CA and pellet chow groups (*P* < .05). Both the degree and time course of the reduction in food intake were dose dependent. The anorexigenic effects of the lowest dose (25 mg/day) were apparent by the end of week 7 (*P* < .05), the intermediate dose (50 mg/kg/day) emerged by the end of week 4, and the effects of the highest dose (100 mg/kg/day) emerged were already evident at the beginning of the third week.

### 3.2. Body Weight

**Figure 2:**
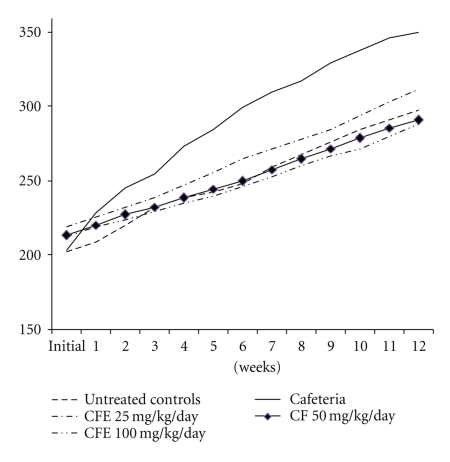
Effect of CFE on rats' body weight in grams over 12 weeks.

Animals in the untreated control group increased body weight by 147.50% (297.66 ± 2.78 g) at the end of the experiment (Figure 2). Those in the CA group increased weight by 172.30% (350.34 ± 2.13 g), significantly more than the untreated control (*P* < .05). Rats in all three CA + CFE groups gained weight at or close to the lower rate of the untreated control group. There was a hint of a dose-response curve. Weight gain in the CA + 25 mg/kg/day CFE group was 142.46% (312.12 ± 3.21 g), CA + 50 mg/kg/day CFE group was 136.53% (291.25 ± 3.11 g), and CA + 100 mg/kg/day CFE group brought to 136.00% (288.50 ± 2.11 g). These weight gains were all significantly less than the CA-fed animals (*P* < .05, Figure 1).50 mg/kg/day of CFE appears to be the optimal antiobesogenic dose.

Table 1 shows that administration of the CA diet significantly increased the weight of perirenal (*P* = .0001), epididymal (*P* = .0001), and mesenteric fat pads (*P* = .0001) compared to the animals fed pellet chow. In animals fed CA + CFE, weight gain in all fat pads was considerably lower than in the CA group, with the 50 and 100 mg/kg/day doses giving results close to the untreated control group. The CA diet resulted in fatty liver and increased the liver mass by 132.66 % compared to the untreated animals (*P* < .0001). In rats given CA + CFE, liver weight gain was reduced in a dose-related manner. At 25 mg/kg/day CFE, liver weight was reduced effectively to the untreated control value, that is, 105.14%. At 50 mg/kg/day CFE, liver weight was below the untreated control value, at 78.57%, and at 100 mg/kg/day CFE liver weight was reduced to 75.14%. The reduced liver weight in the two higher-dose groups reflects the reduced calorific intake and reduced body weight gain at these doses; there were no signs of hepatotoxicity.

#### 3.2.1. Serum Obesity Indicators

The cafeteria diet produced the predicted changes in all serum obesity indicators, all of which were positively modified by CFE in a generally dose-dependent manner, as indicated in Table 2.

Table 2 shows that total cholesterol in the CA group increased by 125.90% (74.78 ± 0.46 mg/dL) compared to untreated controls (*P* < .0001). Coadministration of CFE at 25 mg/kg/day had comparatively little effect (119.26%; 89.19 ± 1.61; *P* = .001); at 50 mg/kg/day the increase was restricted to 110.79% (82.86 ± 1.73; *P* = .025); at 100 mg/kg/day total cholesterol was effectively normalized, at 102.01% (76.30 ± 1.29) with no statistical differences between this group and the controls (*P* = .797).

Serum triglycerides in the CA group increased by 207.76% (150.9 ± 1.61) compared to untreated controls (*P* < .0001). Coadministration of 25, 50, and 100 mg/kg/day CFE restricted this increase to 161.59% (117.30 ± 0.92; *P* = .0001), 149.29% (108.36 ± 1.51; *P* = .0001), and 132.08% (95.9 ± 0.75; *P* = .0001), respectively.

LDL levels in the CA group were significantly increased compared to untreated controls (*P* = .0001). While coadministration of 25 mg/kg/day CFE did not have a statistically significant impact; 50 (*P* = .0001) and 100 mg/kg/day CFE (*P* = .0001) effectively prevented any increase in LDL levels.

Serum HDL levels fell in the CA group compared to untreated controls (*P* = .0001). Coadministration of 50 mg/kg/day CFE progressively reduced the fall in HDL levels (*P* = .001), while 100 mg/kg/day CFE effectively prevented any decline in HDL levels (*P* = .0001).

VLDL levels were significantly elevated in CA-fed rats compared to the untreated controls (*P* = .0001). Coadministration of 25 (*P* = .001), 50 (*P* = .0001), and 100 mg/kg/day (*P* = .0001) CFE significantly inhibited this increase in a dose-dependent manner.

### 3.3. Serum Leptin Concentrations

Table 2 also shows that in the CA group, the serum leptin concentration was dramatically higher than in untreated animals (*P* = .0001). Concurrent administration of CFE effectively prevented this increase (*P* = .0001) at all doses.

### 3.4. Atheroma Formation

**Figure 3:**
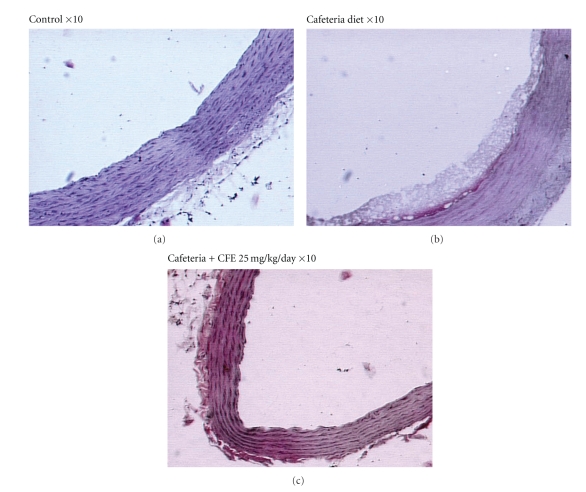
Antiatherogenic assay.

Atheroma formation is the hallmark feature in the development of clinical atherosclerosis.  Figure 3(a) shows a section of healthy aorta taken from a rat fed on lab chow. Feeding rats a cafeteria diet with high fat content caused deposition of lipids in the intimal region of the aortic arch, as shown in Figure 3(b). Treating rats concurrently with different doses of CFE completely prevented lipid deposition and restored normal aortic characteristics as shown in Figure 3(c) which is representative of all three CFE groups.

## 4. Discussion

Feeding rats a cafeteria, high-fat diet inevitably causes hyperphagia resulting in increased body weight compared to pellet chow fed animals and is a widely accepted model for clinical obesity. This gain in body weight is largely due to increased fat mass as a result of preadipocyte proliferation and differentiation and, to an extent, accumulation of lipids in the liver [11, 12]. Our results demonstrate that CFE has pronounced dose-dependent appetite suppressant and antiobesogenic effects in this model. These effects were reflected in the feed intake, body weight, liver weight, fat pad mass, and serum lipid profiles of the rats in our various treatment groups. The hyperleptinaemia and implicit leptin resistance characteristics of obesity were abolished by CFE. Fifty mg/kg/day of CFE appears to be the optimal dose for preventing CA diet-induced changes in body weight, hormones, fat pads, and liver. Kidney and liver function data were assayed at all probe points. Slight negative changes in liver and kidney function induced by the cafeteria diet were reduced by CFE in a dose-dependent manner and approached normal values at the intermediate dose level. The null findings are not detailed here; two clinical trials of the food extract CFE also found no adverse effects [13, 14].

We believe that CFE may act via multiple mechanisms. The decline in food intake may reflect direct intervention in appetite control at the level of the hypothalamus, where the pregnane glycosides are known to act [8]. There is also evidence that the pregnane glycosides act directly on adipose tissue, by inhibiting adipocyte proliferation and differentiation [15–17]. An alternative hypothesis is that CFE may downregulate ghrelin synthesis in the stomach and subsequently neuropeptide-Y in the hypothalamus, with ultimately the same effect of appetite suppression [18–21]. 

Finally, and very much in keeping with the favorable metabolic effects listed above, we showed for the first time that CFE has potent antiatherogenic properties in a rodent model. Concurrent administration with CFE in CA-fed rats completely prevented the accumulation of lipids in the intima of the thoracic aorta, which otherwise develops in these animals [22]. We speculate that the antiatherogenic effects may be mediated at least in part via improved plasma lipid profiles, with additional protection possibly conferred by CFE's antioxidant [23] and anti-inflammatory [24] properties.

## 5. Conclusion

This small scale study suggested that *Caralluma fimbriata* extract showed pronounced dose-dependent appetite suppressant and antiobesogenic effects on a sample of rats fed a cafeteria diet. These data, combined with existing CFE clinical trial findings [13, 14], indicate that CFE has the potential to curb obesity and the pathologies linked to obesity.

## Figures and Tables

**Table 1:** Morphometric analyses of fat pads and liver weight.

Weight in grams	Mean values					*Browne-Forsyth*	Sig.
	Untreated control	Cafeteria	25 mg CFE	50 mg CFE	100 mg CFE		
	(SEM)	(SEM)	(SEM)	(SEM)	(SEM)		
Perirenal	3.36 (±0.11)	11.33 (±0.38)	6.29 (±0.59)	4.83 (±0.21)	3.85 (±0.21)	88.42	0.0001
Epididymal	2.13 (±0.11)	6.4 (±0.38)	4.5 (±0.59)	3.4 (±0.21)	3.9 (±0.21)	51.1	0.0001
Mesenteric	3.52 (±0.17)	6.76 (±0.22)	4.35 (±0.13)	3.18 (±0.24)	2.83 (±0.16)	71.2	0.0001
Liver	10.5 (±0.31)	13.9 (±0.27)	11.04 (±0.36)	8.26 (±0.18)	7.9 (±0.21)	79.3	0.0001
*N* in each group	6	6	6	6	6		

**Table 2:** Obesity indicators and impact of CFE.

Weight in grams	Mean values					*Browne-Forsyth*	Sig.
	Untreated control	Cafeteria	25 mg CFE	50 mg CFE	100 mg CFE		
	(SEM)	(SEM)	(SEM)	(SEM)	(SEM)		
*Serum lipids*							
Cholesterol	74.78 (±0.46)	94.16 (±0.48)	89.20 (±1.61)	82.86 (±1.73)	76.30 (±1.29)	44.63	0.0001
Triglyceride	72.6 (±0.98)	150.9 (±1.61)	117.3 (±0.93)	108.36 (±1.51)	95.9 (±0.75)	568.74	0.0001
LDL	18.89 (±1.02)	36.5 (±1.40)	35.54 (±1.02)	20.37 (±0.79)	14.28 (±1.08)	88.8	0.0001
HDL	42.8 (±1.92)	25.62 (±1.78)	25.5 (±2.3)	40.9 (±0.64)	43.0 (±0.74)	32.15	0.0001
VLDL	13.15 (±0.60)	33.02 (±1.19)	23.57 (±0.8)	21.62 (±0.93)	19.62 (±1.1)	58.01	0.0001
*Serum leptin*	1.65 (±0.17)	13.9 (±0.68)	3.40 (±0.11)	1.92 (±0.20)	1.63 (±0.27)	1237.7	0.0001
*N* in each group	6	6	6	6	6		
